# Functionally Relevant Feeding Enrichment: An Examination of Nectar Feeding Devices With Two Species of Parrot

**DOI:** 10.1002/vms3.71175

**Published:** 2026-08-17

**Authors:** Wing Sum Pang, Yat Sum Man, Eduardo J. Fernandez

**Affiliations:** ^1^ School of Animal and Veterinary Sciences Adelaide University Adelaide Australia

**Keywords:** behavioural welfare, enrichment, parrots, *Polytelis anthopeplus*, Psittaciformes, *Trichoglossus moluccanus*, zoo

## Abstract

**Background:**

Environmental enrichment can promote natural behaviours and improve welfare in captive birds, but its effectiveness may depend on species‐specific ecological and behavioural adaptations, particularly feeding ecology.

**Objectives:**

This study aims to evaluate the effectiveness of naturalistic nectar bucket feeding enrichment devices in two parrot species with contrasting feeding strategies: rainbow lorikeets (*Trichoglossus moluccanus*), which are primarily nectivorous, and regent parrots (*Polytelis anthopeplus*), which are primarily granivorous.

**Methods:**

Forty‐five rainbow lorikeets and 11 regent parrots were observed under two nectar‐feeding conditions: bowl only, where nectar was provided in bowls alone, and bucket + bowl, where nectar was provided simultaneously in bowls and nectar buckets. Behaviour was recorded using instantaneous scan sampling for three 30‐min observation periods (before, during, and after presentation of the experimental condition) and classified into four categories: ground, aerial, bowl, or bucket activity.

**Results:**

Rainbow lorikeets spent significantly more time engaged in aerial activity during both feeding conditions than during the after period. They also interacted more frequently with the nectar bucket and reduced their use of bowls when buckets were available. In contrast, regent parrots showed no significant changes in general behaviour or in their use of bowls or buckets across observation periods or feeding conditions.

**Conclusions:**

The nectar bucket feeding devices effectively promoted species‐typical foraging behaviour in rainbow lorikeets but had little effect on regent parrots, reflecting differences in feeding ecology. These findings highlight the importance of designing enrichment that is tailored to species‐specific ecological and behavioural needs and evaluating enrichment based on both its biological relevance and its overall effectiveness.

## Graphical abstract

Nectar‐based feeding devices increased aerial activity and bucket use while reducing bowl use in rainbow lorikeets but produced little behavioural change in regent parrots. Species‐specific feeding enrichment promotes natural foraging behaviour and may improve welfare in captive nectarivorous parrots.

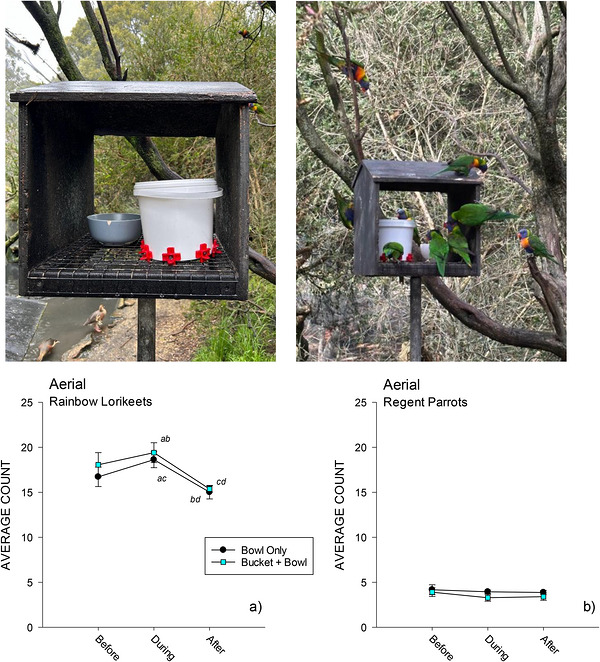

## 1 Introduction

### 1.1 History of Enrichment

The provision of environmental enrichment has become an essential component of modern captivity management, involving the modification of an animal's environment to enhance and maintain both the physical health and psychological well‐being of captive animals by promoting species‐specific behaviours similar to those observed in the wild (Hosey et al. 2013; Kelley and Garcia 2010; Taylor et al. 2023). Historically, enrichment practices emerged from the fields of behavioural engineering and operant conditioning, which were designed to strengthen species‐specific responses through environmental contingencies (Fernandez and Martin 2021; Fernandez 2022). The concept of enrichment gained prominence in the latter 20th century, when animals confined to barren enclosures were observed to develop abnormal behaviours such as pacing and self‐mutilation (Beer et al. 2023; Mason et al. 2007), prompting early pioneers like Desmond Morris to implement feeding devices that encouraged natural foraging behaviours in captive seals (Mellen and Sevenich MacPhee 2001). Likewise, foraging devices have been used extensively to provide for the welfare of zoo birds (Woods et al. 2022).

**Highlights**

- We tested a new nectar bucket feeding device with two different species of parrot.
- Nectar‐feeding rainbow lorikeets showed greater activity and interactions with the bucket devices.
- The mostly granivorous regent parrots showed little change in response to the buckets.
- The functionality of enrichment is largely dependent on the species‐typical repertoires of the animals involved.

### 1.2 Environmental Enrichment

In zoological settings today, enrichment serves as a core welfare strategy that encourages species‐specific behaviours such as foraging, social interaction, and problem‐solving, while reducing abnormal or stereotypic behaviours (Hosey et al. 2013; Swaisgood and Shepherdson 2005). Environmental enrichment is a husbandry approach designed to improve the overall quality of life for captive animals by modifying their surroundings to include beneficial environmental stimuli (Swaisgood and Shepherdson 2005). Enrichment is typically divided into five main categories: food, physical habitat, sensory, social, and cognitive, each addressing various aspects of behavioural and welfare needs (Mellen and Sevenich MacPhee 2001; Young 2003). Food enrichment increases the effort required to obtain food, for example, through hidden treats or puzzle feeders, while physical (habitat) enrichment introduces varied structures to encourage exploration and movement (Bloomsmith et al. 1991; Davison et al. 2024). Sensory enrichment alters visual, olfactory, or auditory stimuli to enhance environmental perception, and social enrichment promotes interaction with conspecifics, though it can also introduce risks, such as stress or aggression (Skibiel et al. 2007; Young 2003). Cognitive enrichment provides mental challenges that stimulate problem‐solving and learning (Clark 2017; Hoy et al. 2009; Meehan and Mench 2007).

### 1.3 Wild and Captive Foraging Behaviour in Parrots

An effective enrichment strategy integrates both the species’ evolutionary background and the individual's learning experiences to create environments that support natural behaviours such as foraging (Fernandez and Martin 2021; Newberry 1995). In the wild, parrots spend a substantial proportion of their active time, often between 40% and 75%, searching for, selecting, and manipulating food items (Meehan et al. 2003; E. L. Mellor et al. 2021; van Zeeland et al. 2013). In captivity, food for parrots and other birds is often delivered in predictable locations and easy‐to‐consume forms, drastically reducing the time spent on natural foraging activities, leading to reduced activity and the potential development of stereotypic behaviours (Davison et al. 2024). To counteract this, enrichment strategies focus on varying feeding methods, such as scattering, hiding, or making food retrieval more challenging (Young 2003). These methods mimic natural foraging and prevent boredom or frustration, thereby improving the overall welfare of captive birds, including parrots (Brereton and Rose 2022).

Rainbow lorikeets (*Trichoglossus moluccanus*) possess a specialized brush‐tipped tongue with elongated thread‐like papillae adapted for efficient nectar and pollen collection, enhancing their ability to extract liquid or soft foods (Kalmar et al. 2009; Rosa 2019). They are competitive and opportunistic feeders that actively compete for resources and exploit diverse food sources rather than relying on a single one (Higgins et al. 1999; Rosa 2019). In South Australia's temperate regions, rainbow lorikeets primarily consume nectar and pollen from the outer foliage of *Eucalyptus*, *Banksia*, *Melaleuca*, *Callistemon*, and *Grevillea* species, along with fruits, seeds, and small invertebrates, which form minor components of their diet (Chapman 2005; Kalmar et al. 2009). Their nectarivorous specialization is reflected in distinct digestive adaptations, including reduced gizzard musculature, thinner koilin layers, and shorter intestinal tracts compared to granivorous parrots (Kalmar et al. 2009). In captivity, nectarivores are typically fed commercially formulated nectars supplemented with fruits (either cubed or blended), occasional insects for protein, and extruded pellets to mimic natural dietary diversity (Cabana and Lee 2018). As such, nectarivores such as rainbow lorikeets could benefit from foraging enrichment devices that allowed them to manipulate the devices with their tongues in order to obtain food.

In contrast, regent parrots (*Polytelis anthopeplus monarchoides*) are predominantly granivorous, feeding on seeds, flowers, and buds (Schultz 2006). Their robust beaks and well‐developed tongue musculature facilitate the de‐husking and processing of seeds and nuts (Kalmar et al. 2009). Regent parrots exhibit flexible foraging strategies, feeding in pairs or small parties both on the ground and in tree canopies (Beardsell and Campbell 1985; Burbidge 1985; Forshaw and Cooper 2002). Their foraging activity spans diverse habitats, including native mallee vegetation, vineyards, orchards, and cereal crops, demonstrating considerable dietary flexibility (Burbidge 1985).

Foraging enrichment has been shown to be effective in enhancing captive parrot welfare (Meehan et al. 2003; Rodríguez‐López 2016). For instance, Coulton et al. (1997) demonstrated that parrots chose to work for food even when identical free food was available, a phenomenon known as contra freeloading, and that providing enrichment can increase positive social behaviours such as allopreening. In captivity, animals are provided with consistent food, water, and shelter, but the lack of environmental complexity restricts opportunities for natural behaviours like flight, exploration, and species‐specific foraging (Inglis et al. 1997; Ogura 2011; Stevens et al. 2021). For rainbow lorikeets, this often means limited use of their brush‐tipped tongues for nectar extraction, a key component of their wild foraging ecology (Rosa 2019). As previously noted, addressing this limitation through food‐based enrichment that mimics natural foraging behaviour can fulfil intrinsic behavioural needs that would otherwise go unmet in captivity, therefore improving animal welfare.

### 1.4 Aim

Despite growing interest in zoo animal welfare, research remains disproportionately focused on mammals, which constitute approximately 75% of all zoo welfare studies, while avian welfare literature accounts for only around 10% (Binding et al. 2020; Woods et al. 2022). This imbalance highlights a significant gap in understanding the welfare needs of captive birds. In particular, limited attention has been given to the development and assessment of environmental enrichment strategies tailored to the species‐specific behaviours of nectarivorous birds such as rainbow lorikeets.

The present study therefore aimed to address this research gap by introducing and evaluating a bucket feeding enrichment device that could support the natural foraging behaviour of captive rainbow lorikeets. Specifically, it sought to compare behavioural responses between rainbow lorikeets and regent parrots and assess whether the enrichment promoted species‐typical feeding, particularly in nectarivorous rainbow lorikeets. It was hypothesized that rainbow lorikeets would exhibit greater interaction and engagement with the enrichment compared to regent parrots, reflecting their natural feeding ecology.

## 2 Material and Methods

### 2.1 Study Site and Subjects

The study was conducted in the swamp aviary at Cleland Wildlife Park, South Australia, Australia. The aviary measures approximately 700 m^2^, being 40 m long, 17 m wide, 10 m high at its highest point, and around 2–3 m high around the edges. The aviary houses a diverse community of birds, including approximately 45 rainbow lorikeets (*T. moluccanus*) and 11 regent parrots (*Polytelis anthopeplus*), which were the focal species of this study. Other species present included three Australian blue‐billed ducks (*Oxyura australis*), two bush stone‐curlews (*Burhinus grallarius*), one eastern whipbird (*Psophodes olivaceus*), 12 glossy ibises (*Plegadis falcinellus*), four pied stilts (*Himantopus leucocephalus*), three plumed whistling ducks (*Dendrocygna eytoni*), eight radjah shelducks (*Radjah radjah*), four royal spoonbills (*Platalea regia*), two white‐faced herons (*Egretta novaehollandiae*), and one yellow‐tufted (helmeted) honeyeater (*Lichenostomus melanops*). The aviary was well planted and naturalistic, containing tree species including bottlebrush (*Callistemon* sp.), boobialla (*Myoporum* sp.), eucalyptus (*Eucalyptus* sp.), and tea tree (*Leptospermum* sp.), along with groundcover and aquatic plants. Most of these plants were not observed to be directly fed upon by either focal species during the study.

### 2.2 Study Materials

**FIGURE 1:**
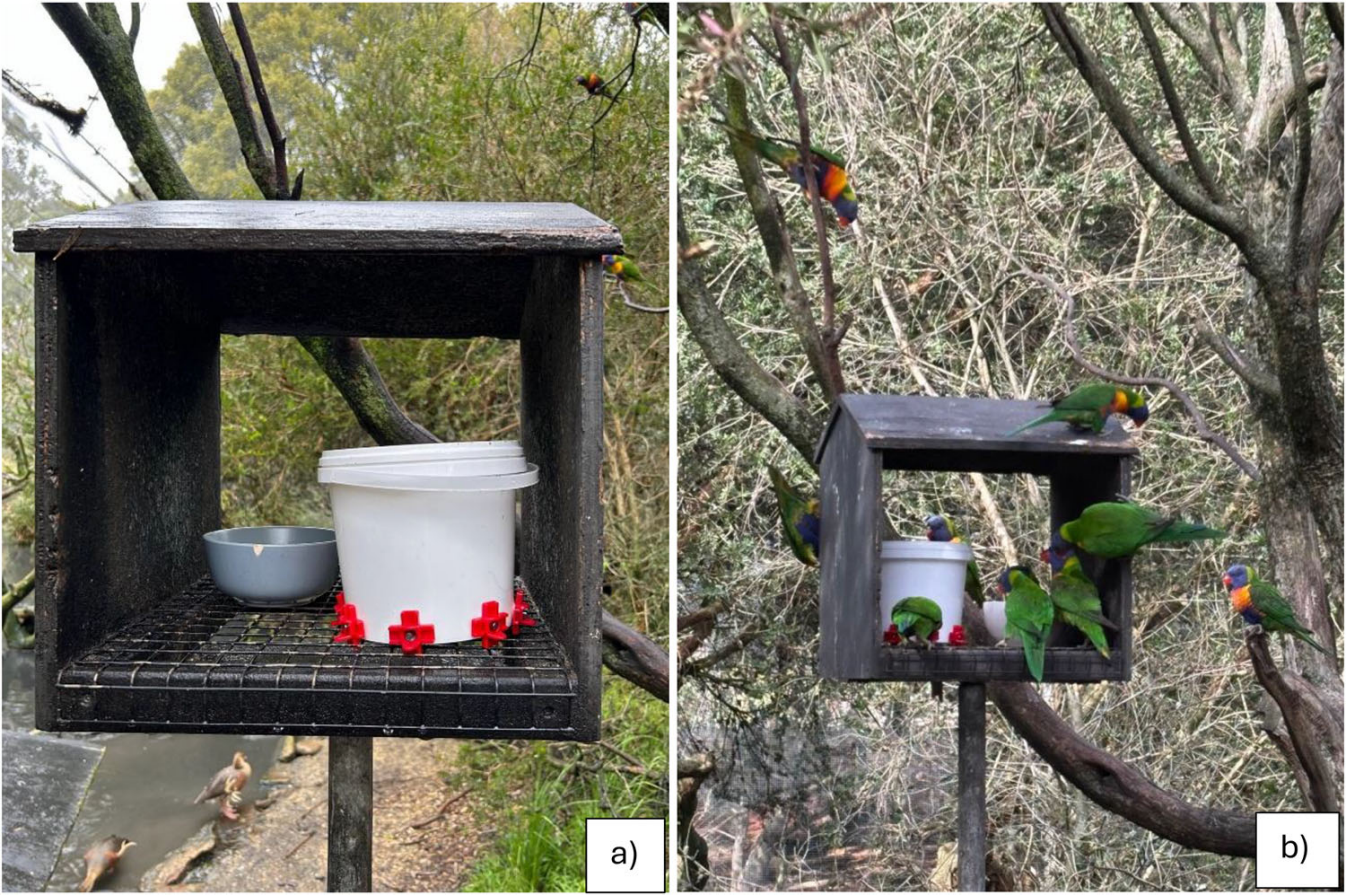
Feeding devices used in the study (a) along with feeding interactions (b). The blue ceramic bowl (left in (a)) represented standard feeding, while the white bucket with eight red nipple feeders (right in (a) and left in (b)) represented the bucket feeding enrichment. Both devices were placed in a sheltered feeding stand within the swamp aviary.

Materials included three ceramic bowls measured 15 cm in diameter (10 cm height; 1.1 L capacity) containing wet nectar that were provided twice daily. Bucket feeding devices used in the study were made from three 2.5‐L white plastic buckets (20 cm diameter; 20 cm height), each fitted with eight nipple feeders positioned around the bottom edge. Three buckets were placed at the three feeding stations next to the ceramic feeding bowls to encourage natural foraging behaviours and increase spatial complexity. Nectar was supplied in equal amounts (∼1 L) to both bowl only and bucket plus bowl conditions. Three feeding stands, each with a 40 × 40 cm platform, were used to support this feeding setup (see Figure 1a,b).

### 2.3 Diet, Supplementation, and Enrichment

The standard diet of rainbow lorikeets included commercially prepared nectar which was used throughout the entire study period, and supplementary dry feed (see details below). They were provided with ‘Wombaroo Complete Lorikeet and Honeyeater Food’ (sucrose, glucose, maltodextrin, whey protein, casein, lysine, methionine, mannan oligosaccharides, β‐glucans, vegetable oils, omega‐3 and omega‐6 fatty acids, taurine, carotenoids, vitamins A, B1, B2, B3, B5, B6, B9, B12, C, D3, E, and K, biotin, choline, inositol, calcium, phosphorus, potassium, sodium, magnesium, zinc, iron, manganese, copper, iodine, and selenium) from Wombaroo Passwell, Australia, referred to locally as ‘wet nectar’, prepared to standard formulation (300 g powder mixed with 800 mL water). Supplementary feeding included ‘Passwell Complete Lorikeet’ (Sucrose, glucose, processed cereals, soy and whey protein isolates, vegetable oils, omega‐3 and omega‐6 fatty acids, lysine, methionine, carotenoids, vitamins A, B1, B2, B3, B5, B6, B9, B12, C, D3, K, biotin, choline, calcium, phosphorus, potassium, sodium, magnesium, zinc, iron, manganese, copper, iodine, selenium, and 1 g/kg Actigen dried yeast prebiotic) from Wombaroo Passwell, Australia, which is the ‘dry nectar’ given to the rainbow lorikeets ad libitum (given dry; not mixed). Daily enrichment items consisted of spiked fruit, typically comprising quarter pieces of apple or orange, thick slices of alternative fruits, or single grapes. On hot days, birds were occasionally provided with a fruit blend consisting of dry nectar mixed with mashed frozen banana.

Regent parrots were maintained on a specialized diet with a variety of components in accordance with standard aviary feeding protocols. Their regular diet included half a cup of meat mix, cake mix, soaked seed, budgie seed, and one cup of Parrot Pellets from Wombaroo Passwell, Australia, along with fresh greens and fruits. The meat mix consisted of 1 kg kangaroo mince, 300 g Insectivore mix, 300 g meat meal, and four eggs. It could be prepared in two ways: either by adding the dry ingredients and eggs to the minced meat and blending until a crumbly texture formed, or by blending all ingredients together in quarter batches. The cake mix was made from 120 g egg and biscuit or soft finch food combined with 120 g insectivore powder. All mixtures were then blended with water to achieve a moist, crumbly consistency suitable for feeding. The food mixture was placed in three metal feeding trays on the ground, where the birds were fed once daily in the morning.

Irregular food‐based enrichment was provided to all birds in the Swamp aviary, which included browse, flowering plants, and gumnuts from eucalypt and related species, such as *Grevillea*, *Hakea*, *Corymbia*, *Eucalyptus*, tea tree, and *Westringia* species. Birds displayed individual and species‐specific preferences for different plant species and flower colours.

### 2.4 Procedures

For the entirety of the study and for both species, two conditions were compared:

1. Bowl only—nectar presented in bowls only.
2. Bucket + bowl—nectar presented in bowls and in naturalistic nectar bucket feeding enrichment.

Feeding sessions for all birds typically began twice daily at 0900 hours for the morning and 1400 hours for the afternoon, with each session lasting up to 3 h and divided into three observation periods of 30 min each:

1. Before—30 min of observation prior to feeding (morning: between 0800 and 0900 hours; afternoon: between 1300 and 1400 hours).
2. During—30 min of observation during feeding (morning: between 0901 and 1000 hours; afternoon: between 1401 and 1500 hours).
3. After—30 min of observation following feeding (morning: between 1001 and 1100 hours; afternoon: between 1501 and 1600 hours).

Behavioural observations were recorded at 1‐min intervals using instantaneous (pinpoint) scan samples throughout each period, thereby producing 30 recordings per observation period. At each interval, the number of rainbow lorikeets and regent parrots displaying each of four operationally defined and mutually exclusive behavioural categories were recorded based on a predetermined ethogram: ground, aerial, bowl, and on bucket (see Table 1). This was then turned into an average count of birds per species engaged in each class of responses per interval (total species bird count/total observation intervals). Data were recorded digitally using the behavioural observation software ZooMonitor. The two observers (the combined first authors) conducted all observations. They were trained by the supervising author via the ethogram and through observation and software demonstrations. During observations when two observers were present (interobserver agreement), the observers stood at least 1 m apart and did not discuss observations while recording.

**TABLE 1:** Rainbow lorikeets and regent parrots ethogram, depicting four different behavioural categories (aerial, ground, bowl, and bucket), accompanied by the definitions and examples of the behaviour.

Behavioural category	Definition
Aerial	Bird is more than half metre off the ground (i.e., in the air, on branch, or on a fence).
Ground	Bird is on or near (within half a meter) of the ground
Bowl	Bird is on a food bowl (three bowls total).
Bucket	Bird is in contact with bucket feeding enrichment (three devices total).

*Note*: Each response was mutually exclusive for any individual bird.

The 8‐week study followed a within‐subject reversal design, alternating between control (A; bowl only) and enrichment (B; bowl + bucket) conditions in the following sequence: ABBAABBA. This resulted in 87 total sessions across the 8 days of observation (42 bowl only sessions; 45 bucket + bowl sessions), with 26 before sessions, 32 during sessions, and 29 after sessions for a total of 43.5 h of observation. The entire study ran once a week, from 22 August, 2025, to 14 October, 2025 (8 days). Two researchers conducted all the observations. All study subjects remained in the study area for the entire study period.

### 2.5 Interobserver Agreement

The raw data were exported from ZooMonitor and compiled in Microsoft Excel, which was then used to calculate all descriptive statistics, including interobserver agreement (IOA; Cooper et al. 2020). IOA was calculated for four of the eight responses across the two species where two observers were present for more than 20% of the sessions (rainbow lorikeet aerial, rainbow lorikeet ground, rainbow lorikeet bowl, regent parrot aerial), resulting in 20 of 87 sessions (23%) of all sessions being assessed. The average IOA was calculated at 81.6% using the following total agreement equation:

(1) IOA=(IOAsessions/totalsessions)×100%

### 2.6 Statistical Analysis

SigmaPlot (version 12.5; Systat Software Inc., San Jose, CA, USA) was used to create all graphs and run all the statistical analyses. No clear differences in morning versus afternoon sessions were observed, and therefore not utilized for statistical comparisons. A 2 × 3 factorial (two‐way) analysis of variance (ANOVA) was used to compare differences for each species and the four broad behavioural categories (eight total comparisons) across two independent variables: condition (bowl only and bucket + bowl) and period (before, during, and after). Although the data for almost all measures failed the Shapiro–Wilk normality test (only rainbow lorikeet Aerial responses passed normality; *p* = 0.422), and all tests failed Brown–Forsythe equal variance tests, a parametric ANOVA approach was implemented due to the robustness of ANOVA to violations of normality with our large sample size (*N* = 87 observations). This approach is consistent with recommendations that ANOVA remains valid with large datasets even when normality assumptions are not fully met (Ghasemi and Zahediasl 2012). When significant main effects or interactions were detected (*p* < 0.05), post hoc tests using the Holm–Sidak method were conducted to identify which groups differed significantly from one another. Differences of means comparisons were calculated to quantify the magnitude of differences between the groups.

## 3 Results

### 3.1 Aerial Activity

**FIGURE 2:**
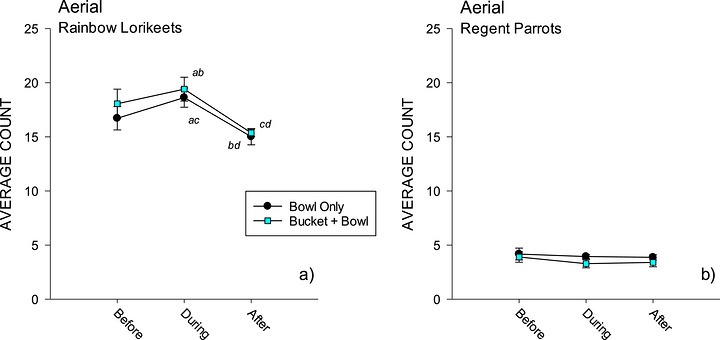
Average count (plus standard error of the mean [SEM] bars) of the aerial behaviour of rainbow lorikeets (a) and regent parrots (b) across three periods (before, during, and after) and two conditions (bowl only and bucket + bowl) for the total 87 sessions of observation. Significant differences (*p* < 0.05) are denoted by differing letters, where identical letters (e.g., a–a) or a lack of letters indicate no significant difference.

Figure 2 presents the average count of aerial behaviour observed (with standard error of the mean [SEM] bars) for (a) rainbow lorikeets and (b) regent parrots across the three observation periods (before, during, and after) under two conditions: bowl only and bucket + bowl. There was a statistically significant difference for the rainbow lorikeets (*F*_2,81_ = 8.213, *p* < 0.001). Post hoc tests revealed that the during periods as a whole were significantly different from the after periods (*t* = 4.047, *p* < 0.001). During periods were also significantly higher compared to the after period for both the bucket + bowl (*t* = 3.054, *p* = 0.009) and the bowl only (*t* = 2.673, *p* = 0.027) conditions. No significant differences were observed for the regent parrots, with the average aerial responses across all conditions and periods occurring below 5. In terms of the descriptive statistics, aerial activity was the highest during the feeding period for both conditions, with the bucket + bowl condition slightly higher (*M* = 19.413, SEM = 0.922) than the bowl only condition (*M* = 18.624, SEM = 0.922). These values for both conditions were greater than those recorded in the before period (bowl only: *M* = 16.723, SEM = 1.064; bucket + bowl: *M* = 18.058, SEM = 0.985) and the after period (bowl only: *M* = 15.019, SEM = 0.985; bucket + bowl: *M* = 15.367, SEM = 0.952).

### 3.2 Ground Activity

**FIGURE 3:**
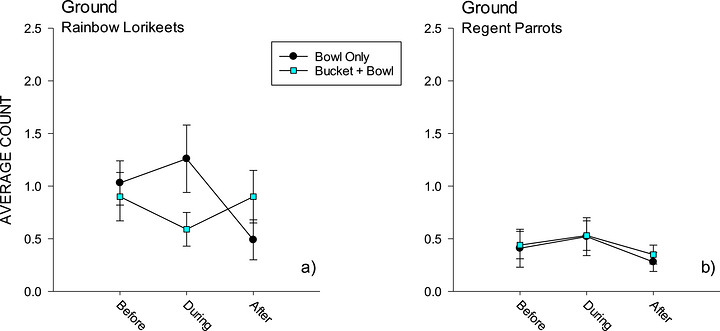
Average count (plus standard error of the mean [SEM] bars) of the ground behaviour of rainbow lorikeets (a) and regent parrots (b) across three periods (before, during, and after) and two conditions (bowl only and bucket + bowl) for the total 87 sessions of observation. Significant differences (*p* < 0.05) are denoted by differing letters, where identical letters (e.g., a–a) or a lack of letters indicate no significant difference.

Figure 3 displays the average count of ground behaviour observed (with standard error of the mean bars) for (a) rainbow lorikeets and (b) regent parrots across all three periods (before, during, and after) under two conditions: bowl only and bucket + bowl. No significant differences were observed for either the rainbow lorikeets or the regent parrots. The average ground activity level of rainbow lorikeets occurred at less than 1.5 individuals observed across all conditions and periods, while for the regent parrots, it occurred less than one individual observed across all conditions and periods.

### 3.3 Bowl Activity

**FIGURE 4:**
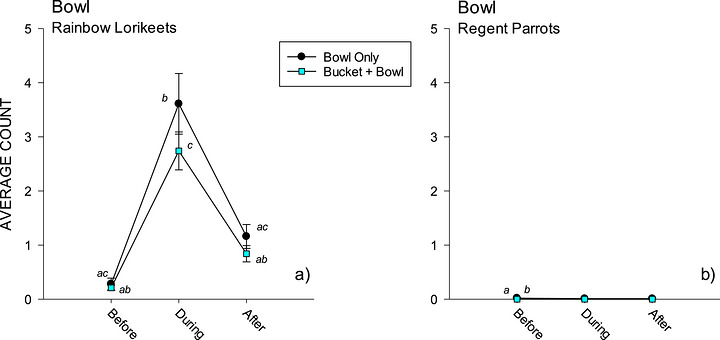
Average count (plus standard error of the mean [SEM] bars) of the bowl‐directed behaviour of rainbow lorikeets (a) and regent parrots (b) across three periods (before, during, and after) and two conditions (bowl only and bucket + bowl) for the total 87 sessions of observation. Significant differences (*p* < 0.05) are denoted by differing letters, where identical letters (e.g., a–a) or a lack of letters indicate no significant difference.

Figure 4 presents the average count of bowl activity observed (with standard error of the mean bars) for (a) rainbow lorikeets and (b) regent parrots across the three observation periods (before, during, and after) under two conditions: bowl only and bucket + bowl. There was a statistically significant difference for the rainbow lorikeets (*F*_2,81_ = 45.735, *p* < 0.001). Post hoc tests also revealed that the periods as a whole were all significantly different from each other (during vs. before: *t* = 9.052, *p* < 0.001; during vs. after: *t* = 6.911, *p* < 0.001; after vs. before: *t* = 2.297, *p* = 0.024). Overall, interaction with the bowl was lower in the bucket + bowl condition compared with the bowl only condition. During the feeding period, bowl activity in the bowl only condition (*M* = 3.607, SEM = 0.306) showed a higher average count than in the bucket + bowl condition (*M* = 2.744, SEM = 0.306), which was also significantly different (*t* = 1.995, *p* = 0.049). The during periods were also significantly higher compared to the before period for both the bucket + bowl (*t* = 5.664, *p* < 0.001) and the bowl only (*t* = 7.109, *p* < 0.001) conditions. Finally, the during periods were significantly higher compared to the after period for both the bucket + bowl (*t* = 4.314, *p* < 0.001) and the bowl only (*t* = 5.449, *p* < 0.001) conditions. For regent parrots, there was also a statistically significant difference (*F*_1,81_ = 4.732, *p* = 0.033). Their bowl interaction remained generally low (approximately 0 for all periods and conditions), but there was a statistically significance difference between the bowl only and bucket + bowl (*t* = 2.102, *p* = 0.039) conditions, with bowl activity being slightly higher in the before period for the bowl only condition compared to the bucket + bowl condition.

### 3.4 Bucket Activity

**FIGURE 5:**
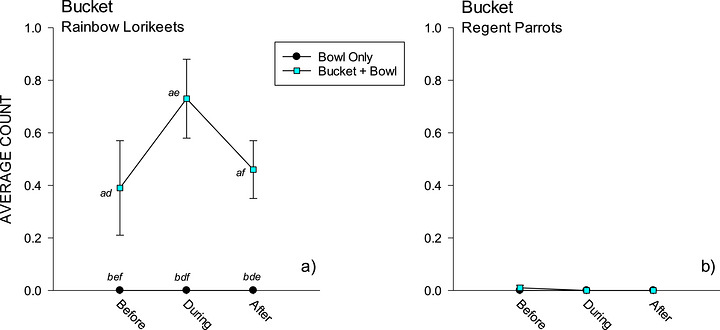
Average count (plus standard error of the mean [SEM] bars) of the bucket‐directed behaviour of rainbow lorikeets (a) and regent parrots (b) across three periods (before, during, and after) and two conditions (bowl only and bucket + bowl) for the total 87 sessions of observation. Significant differences (*p* < 0.05) are denoted by differing letters, where identical letters (e.g., a–a) or a lack of letters indicate no significant difference.

Figure 5 presents the average count of bucket activity observed (with standard error of the mean bars) for (a) rainbow lorikeets and (b) regent parrots across the three observation periods (before, during, and after) under two conditions: bowl only and bucket + bowl. For rainbow lorikeets, there was a statistically significant difference between the two conditions (*F*_1,81_ = 35.289, *p* < 0.001). Post hoc tests revealed that, as a whole, the bucket + bowl condition produced significantly greater bucket activity than the bowl only condition (*t* = 5.940, *p* < 0.001). Bucket activity was the greatest in the during period (*M* = 0.732, SEM = 0.103), followed by the after period (*M* = 0.462, SEM = 0.107) and the before period (*M* = 0.391, SEM = 0.110). In each case, bucket activity was significantly greater in the bucket + bowl condition compared to the bowl only condition within each period (before: *t* = 2.409, *p* = 0.018; during: *t* = 5.017, *p* < 0.001; after: *t* = 3.010, *p* = 0.003). No significant differences were observed for the regent parrots, with near zero bucket activity recorded in all periods and conditions.

Table 2 lists the average percentage of occurrence (with standard error of the mean; SEM) for the rainbow lorikeets in the before, during, and after periods for the entire 8 weeks of study. The table compares differences between the two conditions (bowl only and bucket + bowl) across the three session periods (before, during, and after). Responses for the four behaviours analyzed are averaged across both of the conditions. Aerial behaviour increased in the during and after period for bucket + bowl condition compared with bowl only condition. Interaction with the bowl decreased in the during and after period for bowl only condition compared with bucket + bowl condition. Interaction with the bucket feeding enrichment increased most in the during compared with after period.

**TABLE 2:** Mean number of occurrence (with standard error of the mean; SEM) for rainbow lorikeets in the before, during, and after periods for bowl only condition and bucket + bowl condition.

Period	Condition	Aerial	Ground	Bowl	Bucket
Before	Bowl only	17.39 (0.73)	1.07 (0.13)	0.28 (0.11)	0.00 (0.00)
	Bucket + bowl	16.72 (1.03)	0.93 (0.12)	0.28 (0.11)	0.39 (0.18)
During	Bowl only	18.62 (0.92)	1.31 (0.26)	3.61 (0.31)	0.00 (0.00)
	Bucket + bowl	19.41 (0.92)	0.63 (0.13)	2.74 (0.35)	0.73 (0.15)
After	Bowl only	15.02 (0.99)	0.49 (0.19)	1.17 (0.22)	0.00 (0.00)
	Bucket + bowl	15.37 (0.95)	0.85 (0.15)	0.94 (0.22)	0.46 (0.11)

*Note*: Results are presented for rainbow lorikeets across the three periods and two conditions: bowl only and bucket + bowl, and for the four most frequent behaviours (aerial activity, ground behaviour, bowl usage, and bucket usage) measured.

## 4 Discussion

### 4.1 Main Findings

The rainbow lorikeets increased their use with the bucket feeding devices and decreased their use with the bowls when the bucket feeding devices were present during all three periods (before, during, and after). In addition, both feeding devices produced significant increases in rainbow lorikeet aerial activity when comparing each condition's during and after periods, with the addition of the buckets producing greater aerial activity compared to the bowls alone, albeit a non‐significant increase. This behavioural preference may reflect the motivation of rainbow lorikeets to engage in nectar foraging activities (van Zeeland et al. 2023). These similar patterns were not observed in the granivorous regent parrots, who showed little change as a result of either nectar feeding options (bowls or buckets). Lorikeets are highly exploratory, nomadic species characterized by a strong interest to interact with novel stimuli which, according to Mettke‐Hofmann (2000), makes them particularly susceptible to developing abnormal behaviours, including feather plucking, when deprived of adequate environmental stimulation. As such, providing nectar‐based food within interactive enrichment devices to rainbow lorikeets appears to increase overall foraging, also possibly stimulating problem‐solving activity, which is an important component of welfare in captive birds, particularly for psittacines (Baukhagen and Engell 2022).

The presence of the bucket feeding device not only increased its direct use by the rainbow lorikeets but concurrently reduced interaction with the feeding bowls, indicating its effectiveness in being used as an environmental enrichment device. Preference itself can play a critical role in the selection and ability of devices to function as enrichment, thereby suggesting preference‐based welfare benefits (Fernandez and Timberlake 2019; Turner et al. 2025). This observed behaviour also suggests that the bucket feeding devices likely alter how rainbow lorikeets interact with their feeding environment, thereby promoting diversification of feeding behaviours. Such changes in behaviour are important in the determination of effective enrichment, as it is often reflected by providing sensory stimulation to increase behavioural diversity and promote positive utilization of the environment while reducing behavioural monotony (Veissier et al. 2024). Overall, the rainbow lorikeets showed the greatest changes to the introduction to the buckets through increased aerial and bucket activity and decreased bowl interactions. Specifically, aerial behavior was consistently higher in the bucket plus bowl condition compared to the bowl only condition across all three periods of observation. The increase in aerial activity, albeit non‐statistically significant, is still a core behaviour of birds, as well as a critical indicator of good welfare in captive environments (Brereton et al. 2025; E. Mellor et al. 2018). Brereton et al. reported that in conditions of abundant food availability, flight may contribute to energy regulation by facilitating expenditure of excess energy, while also enabling birds to exert spatial choice within their environment. Although flight is not essential for survival in captivity, opportunities to perform this behaviour remain important for maintaining normal behavioural repertoires. The Five Freedoms and Five Domains as welfare‐based models also emphasize the importance of natural behavioural expression (D. J. Mellor et al. 2020; Miller and Chinnadurai 2023). Flying is quintessential natural behaviour that is vital for birds to engage in exploration and foraging (Brereton et al. 2025; Kaplan 2022). Conversely, the deprivation of flight opportunities can elicit undesirable consequences, with research demonstrating that reduced activity levels correlate with behavioural problems, including feather plucking and self‐mutilation (Jenkins 2001; Peng et al. 2013).

Increased aerial behaviour in rainbow lorikeets may reflect enhanced foraging activity, as natural foraging involves frequent flight between dispersed food resources (Ruaux et al. 2020). This is supported by Beekmans et al. (2023) who demonstrated that enrichment devices increase daily foraging time in grey parrots by engaging both the appetitive and consummatory phases of foraging behaviour. Similarly, rainbow lorikeets in our study showed sustained engagement with the enrichment device even when their normal feeding bowls were available, along with increased aerial behaviour suggesting increase foraging activity. Similar patterns have been reported by van Zeeland et al. (2013), showing African grey parrots provided with foraging devices spent two times longer foraging compared to their normal feeding conditions. Such enrichment‐driven increases in foraging activity have been widely associated with reduction in stereotypic behaviour and improved welfare (Beekmans et al. 2023; Chalmers et al. 2024; Meehan et al. 2003). Therefore, providing enrichment devices that allow natural foraging is essential for enabling species‐typical behavioural expression and supporting good welfare outcome over the long term.

In contrast, regent parrots showed little interaction with the buckets, which was likely due to differences in feeding ecology, as they are primarily ground‐foraging granivores (Kalmar et al. 2009). Although this absence of response might superficially appear unremarkable, their behavioural consistency across all conditions established a reliable baseline, on par with the broader argument that null results are equally critical for identifying species‐typical patterns and should not be dismissed as failed outcomes (Schweinfurth and Frommen 2025). They additionally helped demonstrate that the bucket did not elicit a curiosity response across all parrot species housed in similar conditions. Finally, this provides additional support that the effectiveness of enrichment is predicted by how closely a device aligns with a species' natural foraging ecology (E. L. Mellor et al. 2021; Rodríguez‐López 2016). Therefore, the regent parrots’ disengagement from the buckets provided additional evidence that the lorikeets' behavioural changes were driven by species‐specific motivation rather than circumstantial factors.

### 4.2 Limitations

One of the main challenges encountered during the study was the limited number of observers. As described in the Materials and Methods section, only two researchers participated throughout the entire data collection period, despite the study involving more than 50 birds distributed across three separate feeding stations. The constant movement reduced the likelihood of consistently recording all behaviours within each interval, particularly when multiple individuals were feeding simultaneously at various locations. Consequently, some behavioural events may have been underreported or missed, potentially affecting the accuracy and completeness of the data set. Although our interobserver agreement/interrater reliability remained above 80%, a greater number of observers or observation methods (e.g., camera recordings) could have increased the reliability and accuracy of our recordings.

Another limitation of the study was the relatively short eight‐week observation period. Although sufficient to capture general behavioural trends and initial responses to the enrichment device, this timeframe may not have allowed for the detection of more subtle or long‐term effects. Behavioural adaptation to enrichment (i.e., habituation) often occurs gradually as individuals become familiar with the new device, develop preferences, or adjust their interaction patterns based on experience (Bulla et al. 2024; Meyers‐Manor 2016). Therefore, short‐term observations may overrepresent initial novelty responses while underestimating sustained behavioural engagement once the enrichment becomes a regular part of the environment. In addition, behavioural expression in captive birds can vary with numerous external factors such as season, temperature, visitor density, breeding condition, and individual differences (Rose et al. 2020; Rose et al. 2021; Xie et al. 2017), and an eight‐week window may fail to capture the influence of these natural fluctuations. For instance, during cooler or less crowded periods, birds may allocate more time to foraging or exploration compared to warmer or busier periods (Blanchett et al. 2020; Mainwaring et al. 2021).

### 4.3 Future Directions

Future studies should continue to explore species‐focused foraging‐based enrichment, such as nectar feeding devices for nectarivores, to better understand their long‐term effects on behaviour and welfare in birds. Longer periods would allow researchers to determine whether the behavioural changes observed persist over time or are influenced by habituation (Meyers‐Manor 2016). Importantly, enrichment should be functional and aligned with species‐typical behaviours, rather than superficial additions that may be mistakenly considered enrichment, such as mirrors in a captive environment, which often fail to provide meaningful behavioural or cognitive stimulation (Brereton et al. 2021).

Additionally, individual and social factors, including potential food resource competition, provides an interesting avenue for future examinations of enrichment introductions that involve group‐based foraging devices. Early in the history of environmental enrichment development, Markowitz (Markowitz 1978, 1982) spent considerable time discussing how such devices and events would impact social interactions. In addition, a recent quantitative review has examined how differences in social introductions have been studied in zoos (Martinez et al. 2025). The combination of research examining enrichment with differences or changes in social dynamics, particularly among taxa with specific social group needs and grouping possibilities such as psittacines, would allow zoo researchers to attend to important welfare variables that likewise could directly aid the management of exhibited animals.

The findings from this study also provide a valuable foundation for future research by demonstrating that bucket feeding device can elicit species‐typical foraging behaviours in rainbow lorikeets. This highlights the potential for practical, welfare‐focused enrichment strategies to be incorporated into routine husbandry practices in captive environments. Future research could build on these results by investigating the cognitive and motivational mechanisms underlying enrichment responses, for example, examining whether such enrichment promotes contra freeloading, where animals work for food despite free alternatives being present (Coulton et al. 1997; Inglis et al. 1997; Ogura 2011). This may enhance the understanding of intrinsic foraging motivation and its link to welfare outcomes. Furthermore, comparing enrichment conditions that require birds to ‘work’ for food, such as nipple feeders or puzzle devices, with freely available food sources could help quantify the degree of engagement and motivation elicited by each condition (Reinhardt and Roberts 1997). Incorporating physiological measures, including stress hormone concentrations or activity levels, alongside behavioural observations would provide a more comprehensive assessment of how such enrichment affects both welfare and overall behavioural expression (de Winkel et al. 2024). Comparative studies across parrot species could further reveal how variations in natural foraging ecology influence enrichment preferences and effectiveness.

## 5 Conclusion

This study demonstrated that using nectar‐based bucket feeding devices could serve an enriching function, as evidenced by increased device usage and aerial activity in rainbow lorikeets. The lorikeets spent less time at the bowl and more time interacting with the buckets, suggesting a preference for feeding opportunities that align with their natural foraging patterns. The study also suggests that foraging‐based enrichment could assist in welfare assessment and potential improvement for captive nectarivorous parrots. Future research should explore long‐term effects (i.e., habituation), cognitive and motivational mechanisms, and species‐specific responses to enrichment. Incorporating physiological (e.g., cortisol) and physical (e.g., body condition scores) measures along with standard ethograms extended observation periods that could additionally utilize effects observed through physiological/physical measures, and comparative studies across parrot species will strengthen our understanding of enrichment effectiveness and its contribution to welfare.

## Author Contributions

**Wing Sum Pang** and **Yat Sum Man**: conceptualization, data curation, formal analysis, investigation, methodology, writing – original draft, writing – review and editing. **Eduardo J. Fernandez**: conceptualization, data curation, formal analysis, investigation, methodology, project administration, software, supervision, writing – original draft, writing – review and editing.

## Funding

The authors have nothing to report.

## Ethics Statement

The research was conducted in accordance with ethical standards for animal care and welfare and was approved by the University of Adelaide Animal Ethics Committee (S‐2025‐0889) and exempted by the Wildlife Ethics Committee of the Department for Environment and Water (37/2025).

## Conflicts of Interest

The authors declare no conflicts of interest.

## Data Availability

The data that support the findings of this study are available on request from the corresponding author. The data are not publicly available due to privacy or ethical restrictions.
